# Observations on the Putrid Ulcer

**Published:** 1785

**Authors:** 


VII. *Obſervations on the Putrid Ulcer. Communicated in a Letter to* Samuel Foart Simmons, *M. D. F. R. S. by Mr.* Leonard Gilleſpie, *Surgeon of the Navy, and late Aſſiſtant Surgeon to His Majeſty's Naval Hoſpital at St. Lucia.*

THE putrid or ſcorbutic ulcer proved to be one of the moſt troubleſome, inveterate, and dangerous diſeaſes which afflicted the Britiſh ſeamen employed in the Leeward Iſlands during the late war. The ravages which I have ſeen made by it, as well in naval hoſpitals as on board the fleet; the many opportunities I had of comparing different methods of treatment, and of obſerving the moſt eligible; and the duty I owe to humanity in general, and the naval ſervice of my country in particular, all induce me to lay before the public a few remarks on this complaint.

I prefer the name of putrid ulcer to any other, becauſe the marks of putreſcency always were evident to the ſenſes, although thoſe of ſcurvy were not. It, in general, did not attack the the ſhips crews until they had been at leaſt a year in the Weſt Indies; and it often ſeemed to rage epidemically on board certain ſhips, whilſt others remained, in a great meaſure, free from it: thus His Majeſty's ſhips Ajax, Montagu, Ruſſel, and Triumph, in the beginning of 1781, landed a great number of men on Pigeon Iſland, St. Lucia, with ulcers of the moſt malignant nature, whilſt ſeveral ſhips in the fleet, which had been employed the ſame length of time, in the ſame climate, and on the ſame ſervice, remained, in a great meaſure, free from ſuch complaints. It often affected thoſe who began to recover from fever or dyſentery, and thoſe who had other ſymptoms of ſcurvy; but it often occurred to thoſe who had been healthy during their continuance in the Weſt Indies, and moſt generally after having received a wound or contuſion, however ſlight, particularly of the lower extremities.

The wounds of ſeamen, received in the different general actions, were generally affected with this putrid exulceration, and horrid were the devaſtations made by it. Thoſe who had been formerly affected with ulcers of the legs ſeldom eſcaped a return of their complaint after having been ſome time in the country.

The

The bites of moſchitoes often gave occaſion to this diſeaſe; and ſometimes, without any evident exciting cauſe, a ſmall pimple made its appearance on the leg or foot, which, on being ſcratched, oozed out a ſmall quantity of ſerum; an inflammation of a livid reddiſh colour and diffuſed appearance generally ſucceeded; and when, in this ſtate, warm fomentations and poultices were applied, with a view of diſcuſſing the inflammation, the ulcer ſoon began to ſpread; a fœtid, corroſive ichor was diſcharged, which ſoon acted on the ſurrounding parts, and, in the ſpace of a few days, produced a foul, ſloughy, gangrenous ulcer. A conſiderable degree of fever generally accompanied theſe external appearances, with great thirſt and reſtleſſneſs. It generally happened when the complaint affected the extremities, particularly the lower ones, that the lymphatic veſſels and glands of the part were more or leſs affected with ſwelling and pain; a circumſtance which I have often obſerved even when there was no ulceration, and which ſeems to be meant by Celſus, who ſays, in ſpeaking of fever*, " Igitur ſi ſemel tantum acceſſit,

* Cap. v. lib. 3.

" deinde desiit, eaque vel ex inguine, vel ex

" lassitudine; vel ex æstu, aliave simili re fuit,

" sic ut interior nulla causa metum fecerit,

" postero die, cum tempus accessionis ita tran-

" siit, ut nihil moveret, cibus dari potest."

When the disease attacked the leg, it seldom confined itself to the soft parts. The sloughy, gangrenous disposition frequently affected the periosteum of the tibia, and was accompanied with the most excessive pain; large and profound sloughs were formed, the limb became œdematous, and hæmorrhages often occurred.

In the autumnal months of the year 1780, I had an opportunity of seeing about two hundred cases of scorbutic ulcers in the naval hospital at New York, some of which belonged to the ships of the West-India squadron, which was then at that place in order to avoid the hurricanes; others belonged to the American squadron. The hospital was well provided with every thing necessary, as well of diet as medicine. Every attention was bestowed, in order to keep the sores clean; bark and wine were liberally dispensed, and opium was not sparingly administered; but in vain were cataplasms, fomentations, and warm dressings applied, as they seemed evidently to hasten the rapid progrefs greſs of the diſeaſe. Precipitate powder, which was propoſed and tried as a detergent, produced ſtill worſe effects, and ſimple dreſſings of dry lint were very far from putting a ſtop to the rapid putrefaction. A number of men were rendered unfit for ſervice. Amputation was had recourſe to in ſome inſtances, but with very indifferent ſucceſs, as the ſloughy diſpoſition generally made its appearance on the ſtump; and a great many men loſt their lives by this dreadful diſeaſe, in which they might literally be ſaid to die by inches.

Finding the inefficacy of ſimple dreſſings, and the very bad effects attending the uſe of warm poultices and fomentations, the powder and decoction of bark were tried, but not with any very obvious benefit. Vinegar and water anſwered much better: but what anſwered the beſt of any was, a ſolution of the gummi kino in equal quantities of claret and red port; by the uſe of this the large, fœtid, bloody diſcharge was diminiſhed and corrected, and a ſtop was put to the exulceration.

In the beginning of 1781 a naval hoſpital was eſtabliſhed on Pigeon Iſland, St. Lucia, which was ſoon crowded with patients affected with the moſt dreadful ulcers. The progreſs of the difeafe, as might naturally be expected, was much more rapid in the hotter climate of St. Lucia than at New York; and what tended much to increafe the malignity of thofe complaints was the exceffive fcarcity of all vegetable productions, the hurricane which happened in October of the preceding year having deftroyed them.

The fame plan of treatment was followed here as at New York. Bark, wine, and opium were adminiftered internally in as large quantities as they, perhaps, ever have been adminiftered. A folution of effence of malt was allowed as common drink; but I could not then, nor have I ever obferved, any confiderable effects from that fubftance which was furnifhed to the fhips in the Weft Indies, during the war, at fuch confiderable expence. This I am confident of, that had one half of the money been laid out even on fugar canes, or their juice, they would have been found a much more ferviceable anti-fcorbutic.

The external applications were either warm poultices, or cold fermented ones; lint, dry, or wetted in vegeto-mineral water; pledgets of cerate, &c. We purfued this mode of treatment for fome time, and were inclined to attribute bute the bad fuccefs of it to the want of fruit and vegetables; but when we found thefe were not fufficient to check the rapid progrefs of the difeafe, we gladly purfued a different courfe.

We loft a very confiderable number of men by this difeafe during the firft five months after the eftablifhment of the hofpital. When the complaint affected the leg, its general progrefs was from a trifling fore, often proceeding from a hurt, till it became furrounded with an eryfipelatous inflammation, difcharging an ichorous matter, which darkened the lint applied to it, and afforded the moft fœtid fmell; blood was often difcharged in a thin diffolved ftate, fometimes by an oozing from the entire furface of the ulcer, and at other times from fmall veffels, the mouths of which, though vifible, were with great difficulty clofed, owing to the great degree of putrefraction. There was generally fome pain and tenfion of the inguinal glands; a confiderable degree of fever attended the firft ftage of the complaint, with great thirft; the belly was inclined to be bound at firft, but as the difeafe advanced in its progrefs, a dyfentery or diarrhœa generally made their appearance, and, in the end, carried off the patient. The difeafe was fometimes more rapid in its progrefs, and, in the courfe of a few days, feized on and denuded the tibia, whilft large pieces of integuments and cellular membrane were found to be entirely mortified.

When a flight wound was received in one of the toes of a perfon of a fcorbutic habit of body, it often produced a fpreading floughy ulcer, which very foon affected the bones with caries; and if an attempt was made to put a ftop to the difeafe by amputation of one or more toes, the ulceration and caries generally made a very rapid progrefs, fo as, in fome cafes, to render the amputation of the leg neceffary. I have obferved the fame bad effects from amputation of the fingers in very bad habits, and have always obferved the truth of Mr. Pott's obfervation on the evil effects of applying a cutting inftrument to the toes or fingers in a floughy ftate, whilft one living fibre remained.

Having feen the good effects of vinegar in the naval hofpital at New York, when applied to putrid ulcers, and being convinced, by reafoning from analogy, that the vegetable acid of limes would prove ftill more powerful, I made fome inquiries amongft the negroes employed about the hofpital to know what was their practice in bad fores. I was not greatly furprifed prifed when I learnt that their common practice in putrid fores was to apply thin flices of limes over the furface of the fore, which were repeated two or three times during the courfe of the day.

Mr. Bulcock, furgeon of the hofpital, whom I affifted, readily allowed a trial to be made of this new application. We began by applying a mixture of lime juice and water, in which the lint to be applied was wetted, as were the bandage and comprefs; but we were foon encouraged by its good effects to apply it in a more concentrated ftate, and even to cover the furface of the fores with flices of limes.

The quicknefs with which a ftop was put to large fpreading ulcers was aftonifhing; large mortified floughs were foon caft off; the bloody difcharge ceafed, in general, after the firft application, and the fœtor, which had been infupportable, entirely difappearing, a mild, inodorous pus, with healthy granulations, fucceeded.

We continued to make ufe of limes, and lime juice, as a dreffing in a great variety of cafes:—firft, in putrid, fcorbutic, gangrenous, floughy ulcers, fuch as occur after fevers or other acute difeafes, or take place in bad habits of of body, or in hot countries where the atmoſphere is ſtrongly loaded with marſh miaſmata, or ſuch as are obſerved in hoſpitals that are too much crowded (*la gangrene humide des hopitaux* of the French ſurgeons); for, in my opinion, theſe complaints, though diſtinguiſhed by different names, bear a great reſemblance to each other, and require to be treated in a manner nearly ſimilar.

I think they may all be comprehended under the name of putrid ulcer, from this ſimple and obvious characteriſtic, viz. their yielding a diſcharge always fœtid, often bloody; their having that appearance of ſanies or ichor different from good pus, and which irritates and corrodes, or acts as a ſeptic ferment on the ſurrounding parts, and is often attended with great pain, debility, hectic fever, and a fatal diarrhœa. In all ſuch caſes, I can, from much experience, venture to recommend the application of freſh vegetable acid as a moſt excellent remedy. At this day, when the abſorbent ſyſtem and its functions are ſo well aſcertained, that part of ſurgery which treats of the topical application of medicines (a part ſo much cultivated by the ancient ſurgeons, and ſo little regarded by the more-modern ones) ſeems to regain the importance it ſo highly highly deferves; and happy ſhall I be if the recommendation I give to the vegetable acid, as a topic, ſhould tempt ſurgeons to try the effects of it and other freſh vegetable productions in ſurgical complaints, convinced as I am that the ill ſuccefs of operations in large hoſpitals may, in a great meaſure, be attributed to thoſe putrid ſuppurations which too often baffle the uſe of internal medicines when ſufficient attention is not paid to the topical treatment of the part.

I have daily attended the Hotel Dieu at Paris for upwards of a twelvemonth, and have been witneſs to a vaſt number of operations performed with the greateſt dexterity, yet attended with very bad ſucceſs, for a putrid ſuppuration generally ſucceeded and carried off the patients: and I am inclined to believe that the lives of ſome hundreds might annually be ſaved in that houſe, who fall a ſacrifice to putrid ſuppurations after operations, compound fractures, large abſceſſes, &c. were care taken to correct the putrid fœtor of the ſores by vegetable antiſeptics, particularly the freſh vegetable acid.

It has been alledged by ſome authors, that putrid ſuppurations often prove infectious to patients patients confined with furgical difeafes in the fame wards of hofpitals; and I am much inclined to join them in opinion from what I have obferved in hofpitals; for I have often remarked, that a healthy perfon who had been received for a flight wound, and placed in the furgical ward of an hofpital where there were many patients labouring under putrid fuppurations, has foon been affected in the fame manner; and I have not a doubt that the vegetable acid, by correcting the offenfive fœtor, or by neutralifing it, prevents the fpreading of infection.

Secondly, In ulcers, attended with caries of the bones, the application of lime juice was attended with the moft happy effects, and foon brought about an exfoliation. When the caries extended to a confiderable thicknefs in the tibia, or other bone, perforations were made down to the living parts; flices of limes were then applied over the caries and the furrounding foft parts, with the effect of preventing the fpreading of the caries, repreffing fungous excrefcences, (which, in thefe fort of ulcers, are fo very troublefome, and which we found impoffible to be kept down previous to our purfuing this method of treatment) allaying pain, and preventing hæmorrhage.—We had many inſtances of ulcers with caries of the tibia, of a conſiderable ſize, being caſt off in a very ſhort ſpace of time by this method of treatment. When the caries did not extend to any depth, but affected merely the external lamellæ, no exfoliation took place, the earthy part of the carious bone being found in the form of a blackiſh powder on the dreſſings.

Thirdly, We experienced the moſt deciſively good effects from this application in protracted venereal complaints, particularly in that large, ſpreading, ſloughy ulceration which often ſucceeds the opening of buboes in bad habits, and which often occurs amongſt ſeamen; ſome fatal inſtances of which I have been witneſs to in naval hoſpitals. The dangerous effects of long-continued courſes of mercury, in patients of a bad habit of body, have been very well pointed out by ſome late authors; but if ſuch courſes have been found ſo prejudicial in perſons enjoying their native, healthful air, and every convenience of life, how much more deſtructive muſt they prove to highly-ſcorbutic ſeamen in an unhealthy ſpot between the tropics!—Indeed we admitted ſo many patients at the St. Lucia hoſpital, whoſe conſtitutions had ſeverely ſuffered by a too-long-continued uſe of mercurials, (the effects of which appeared either in ill-conditioned fores of the groin or prepuce; in dyfentery or diarrhœa, attended with hectic fever; or in phthifis pulmonalis, in thofe predifpofed to that complaint) that I am much inclined to believe that the remedy often occafions more evil than the difeafe in that climate, particularly amongft feamen: nor can I too much recommend to thofe who have the care of our brave feamen, to beware how they adminifter mercury, and to be careful in obferving the tendency to fcorbutic diathefis, being convinced that this complication takes place much oftener than is generally fufpected.

As moft of the patients received at the naval hofpital on Pigeon Ifland, for venereal complaints, had previoufly undergone a courfe of mercurials, which had left them in a ftate of debility; and as the appearance of their ulcers, whether of the groin or prepuce, exhibited the fame floughy difpofition above mentioned, we feldom hefitated to lay afide the ufe of mercurials, and proceed to the ufe of tonics, fuch as bark and wine, correcting at the fame time the fcorbutic diathefis by fruit and vegetables, and procuring reft and eafe by the occafional adminiftration of opium. The fores were dreffed dreſſed with ſlices of limes, or their juice, and it had the ſame happy effects in correcting the putrid diſcharge and deterging them. When there was a phymoſis, from ulcers under the prepuce, a mixture of lime juice and water was injected with good effect; and even in gonorrhœa, evidently complicated with ſcurvy, we ſometimes had recourſe to the ſame injection with very good ſucceſs. I am led to believe, from ſome experiments I have made, that a dilute ſolution of the *mercur. calcinat.* or any of the precipitates of mercury, in lime or lemon juice, will be found an excellent application in many venereal caſes, the operation of it being much milder, and, if I do not deceive myſelf, much more efficacious than the mineral ſaline preparations of mercury.

Fourthly, In large abſceſſes we experienced the moſt happy effects from the uſe of this topical application. The danger attending large collections of matter in bad habits of body, particularly when large openings are made, is well known to Engliſh ſurgeons *, and the advantages

* I wiſh the dangerous conſequences attending the making of large inciſions, in order to diſcharge collections of matter, were equally well known to the French ſurgeons, as I am convinced vantages of a feton in fuch cafes are very obvious; but I can venture to recommend, in addition to that practice, the topical application of lime juice. As abfceffes after fevers were very frequent amongft the feamen at St. Lucia, and as they too often continued to difcharge large quantities of pus, fo as to threaten a hectic, and as the furrounding integuments in fome cafes became floughy, we made trial of the lime juice, and found it anfwer very well. When a feton had been introduced, and a large fœtid difcharge was kept up, we moiftened the dreffings with a mixture of the acid and water, and fometimes applied flices of limes to the orifices; but if the abfcefs was not very extenfive, we merely made a depending opening, which we kept from clofing too foon by introducing a fmall tent moiftened with the liquor, over which a comprefs and bandage were applied, moiftened with the fame. The lime juice, by correcting the effufed fluids, procuring a difcharge of mild, laudable pus, and gently ftimulating the parts, foon procured that adhefive inflammation which always takes convinced that a great number of men in the Hotel Dieu of Paris annually fall a facrifice to the cuftom that prevails there of making large incifions.

place place previous to the re-union of ulcerated parts.

We had two or three collections of matter under the *fascia lata* of the thigh, and several others, in different parts of the body, of a very considerable size, successfully treated in this manner; we had also one or two compound fractures, where collections of matter, and a sloughy disposition with a large discharge, made their appearance, treated successfully in the same manner: and as the amputations which were performed in the hospital were done according to Mr. Alanson's method (an account of the great success of which mode of operating will probably be soon published by Mr. Wm. Bulcock, surgeon of the said hospital) when abscesses formed, or a sloughy disposition made its appearance on the stump, we immediately had recourse to limes applied in slices to the external opening, after having injected into the cavity a dilute mixture of lime juice and water; and I am led to believe that some share of the success attending that excellent method of performing amputation, which we so clearly experienced at St. Lucia, may be attributed to this mode of after-treatment; and I cannot help remarking in this place, that

*tetanus*,

*tetanus*, which proved ſo fatal to many brave ſeamen who had limbs amputated after the different general actions in the Weſt Indies, and which carried off at leaſt one half of thoſe operated on in the uſual method, in the commencement of the eſtabliſhment of the hoſpital at St. Lucia, never once made its appearance after the operation was performed after Mr. Alanſon's method — a circumſtance which, in my opinion, more ſtrongly recommends it than any of the other advantages with which it is attended. I can venture to give the ſame ſort of negative recommendation to lime juice as a preventive of *tetanus* in ulcers of the extremities; for though complaints of that ſort often ſucceed very ſlight hurts in the Weſt Indies, and ſeldom fail to prove fatal, yet I have never once known tetanus to come on when lime juice has been applied as a dreſſing to ſores.

In fiſtula *in ano*, after the operation, we experienced the good effects of this application; and in one caſe of fiſtula *in perinæo* I am inclined to believe that a ſtop was put to the diſeaſe, and an adheſion of the parts brought about, by the application of this acid.

Fifthly, We had ſome reaſon to ſuppoſe that the application of the vegetable acid may be frequently ufed with fuccefs in that floughy ftate of the fcalp* which often fucceeds to wounds and contufions of the head, fo fatal in its confequences, and fo well defcribed by Mr. Pott.—A marine belonging to His Majefty's fhip Princefs Amelia, who had been attacked by a number of negroes armed with cutlaffes, and wounded feverely in different parts of the body, was fent to the hofpital in January, 1783. He had more than a dozen cuts on the head in different directions, fome of them penetrating through the firft table; and a triangular piece of the fcalp was perfectly cut off from the reft in fuch a manner that it afterwards floughed off, leaving the cranium bare. His wounds were dreffed in the common method for fome days, until that floughy difpofition of the fcalp, defcribed by Mr. Pott, made its appearance, attended with a great degree of fever, and fome delirium. The pericranium became detached from the bone, and there was every

* That fatal fuppuration of the meninges of the brain, which often follows injuries of the head, is not peculiar to that cavity of the human body; wounds and ulcers of the external parts of the thorax and abdomen often communicate to thofe cavities the fame putrid fuppuration; of which I have feen two or three inftances confirmed by diffection.

ſymptom of miſchief going on under the cranium: whilſt proper attention was given to the treatment by internal medicines, evacuations, &c. his wounds were dreſſed with thin ſlices of limes, over which lint was applied moiſtened with a mixture of lime juice and water, and his bandage was kept conſtantly wet with a ſolution of *ſal. ammon.* in vinegar and water. The good effects of this method of treatment were ſoon remarkable; a ſtop was put to the ſloughy ulceration, the ſores were ſoon deterged, and afforded a laudable matter, and exfoliations took place in a ſhort time.

I could recount a great many more ſurgical complaints in which we uſed lime juice with ſucceſs, as fiſtula lachrymalis, ophthalmias that occur in ſcorbutic perſons, and fœtid ulcers of the ear, in all of which caſes I can venture to recommend a trial of this acid.

It is proper, however, to remark, that though the moſt happy effects were obſervable from the uſe of this external application, yet we did not depend on it alone; as it ſeemed to be leſs efficacious by being conſtantly applied, we intermitted the uſe of it as ſoon as a ſore was properly deterged, and we often made uſe of a poultice made of the recent root of *caſſavi*, or *caſſadu*, grated, grated, in the room of it. Next to lime juice, I know no application equal to that root (the juice of which, taken internally, is well known to prove a deadly poiſon) in bad ſores; and I am well convinced that it would be found exceedingly uſeful in alleviating the pain and correcting the diſcharge of cancers. As it is cultivated in conſiderable quantities in the Weſt-India Iſlands, and as it may be preſerved in ſand for a conſiderable time, the ſurgeons of ſhips in a future war may readily provide themſelves with a ſtock of it when in harbour, and I can well aſſure them it will be found a moſt excellent application. We alſo found good effects from aſtringent applications, ſuch as a ſolution of the *gummi kino*, or *terra Japonica*, in red wine and water, or a decoction of the bark of the cuſſu and mangrove tree, or of the leaves and green fruit of the guava tree, in claret and water. We found ſuch applications neceſſary in that hot climate, where the fibres are ſo much relaxed and the fluids ſo prone to putreſcency. We had alſo recourſe to metallic preparations, as tonics; ſuch as a tincture of braſs in a ſolution of *ſal. ammon.* or a weak ſolution of the vitriol of copper; and we often uſed the Roman vitriol as an eſcharotic.

We kept the patients, as much as poſſible, in an horizontal poſition, when the ulcer affected the leg; and we did not neglect the uſe of bandage made of *bunting*, a thin woollen ſtuff.

With reſpect to the medicines adminiſtered, and the regimen adopted, it may be obſerved, that in the beginning of the complaint, when there was much fever, pain, and reſtleſſneſs, the belly was kept open by laxatives or clyſters; ſaline draughts, made with freſh vegetable acid, were adminiſtered in large doſes; lemonade was allowed as common drink; and opium alone, if the ſtomach was irritable, or joined with ipecacuanha, was given at night, and a perſpiration was encouraged by a draught of warm negus. This had generally the effect of procuring an intermiſſion of the fever, and the bark, in wine, was adminiſtered in ſuch quantities as the ſtomach would bear. The uſe of the bark, however, was not continued uninterruptedly for a ſeries of days, without the moſt urgent neceſſity forced to ſuch a procedure. Experience convinced us that a continued uſe of the bark deprived it of its efficacy; and, on the other hand, we obſerved that there were certain times when the recurrence of fever, and the floughy difpofition of fores, were moft likely to return, that is, about the full and change of the moon. After the authorities cited by Dr. Mead, and many other authors, relative to the influence of the phafes of the moon on difeafes, whether in their attack, courfe, or recurrence; and the notoriety of the fact amongft all people between the tropics, my teftimony can have little weight in this matter in attempting to convince thofe fceptical reafoners, who wifh to deny what has been carefully obferved in different and remote ages, and in different and remote countries, by the favage as well as the philofopher, merely becaufe they are too ignorant of the laws of nature and of the animal œconomy to form a rational theory of the action of lunar attraction on the human body. For my own part, I am fo well convinced of the effects of the phafes of the moon on the human body, pre-difpofed to difeafe, that I think the phyfician fhould not only pay attention to the equinoxes, with Sydenham, but, as the feaman and farmer, taught by experience, expect fome revolution in our atmofphere about the time of the full and change of the moon, fo fhould he expect fome change in the ftate of difeafes. Every perſon who has been affected with an intermittent in an unhealthy ſituation between the tropics, muſt have remarked the regular recurrence of the paroxyſms at the end of a fortnight, anſwering to the full and change of the moon; I have myſelf been obliged to take bark at every full and change for a conſiderable time, at St. Lucia, and I have had occaſion to remark the recurrence of many other diſeaſes, as well as intermittent fevers, at thoſe times.

As to regimen, ſpirituous liquors were totally forbidden, but Teneriffe or Madeira wine was allowed: in the beginning, when a conſiderable degree of fever was preſent, animal food was totally withheld, and afterwards recommended to be ſparingly uſed. Fruit and vegetables of all ſorts were given in as large quantity as they could be procured. The uſe of capſicum, (ſo univerſally uſed by the natives of warm climates) infuſed in vinegar, was often found beneficial in thoſe whoſe ſtomach and inteſtines were weak.

The practice I have now recommended was found to be ſo ſucceſsful, that it was adopted by many of the ſurgeons of the Weſt-India fleet before the concluſion of the war; many of whom, as well as Dr. Blane, phyſician to the the fleet, will, I am ſure, bear teſtimony of the excellent effects of the vegetable acid in ill-conditioned ſores *. It were much to be wiſhed that the navy, eſpecially during the time of war, might be ſupplied with a quantity of the preſerved juice of limes or lemons, an article much more ſalutary as a preventive in fleets and armies than eſſence of malt, or ſtinking ſour krout. That it is poſſible to furniſh a ſufficient quantity of it for the uſe of the fleet, will appear from the following circumſtance: — A Dutch ſhip, from the coaſt of Guinea and Surinam, was captured in the beginning of 1782, and ſent into the Carenage of St. Lucia: ſhe had on board upwards of twelve hundred gallons of lime juice in caſks containing forty gallons each; it had been fined by means of iſinglaſs or the membranes of fiſh, and would have kept, I am inclined to believe, for ſeveral years. We were ſupplied with a quantity of it at the hoſpital on Pigeon Iſland, and found it little inferior to the recent juice.

* Dr. Blane, in a valuable work on the Diſeaſes of Seamen, publiſhed ſince we were favoured with this communication by Mr. Gilleſpie, confirms the account given by the latter of the great efficacy of the juice of limes and lemons as an external application in ſcorbutic ulcers. — EDITOR.

I have fince been informed by a gentleman who had long refided on the coaft of Guinea, that every Dutch factory is obliged to fend home, annually, a certain quantity of the preferved juice of limes. Such a regulation in the Britifh factories might be readily eftablifhed, and would furnifh to our fleets the moft noble prefervative againft thofe difeafes which too often unman them.

When limes, lemons, or their preferved juice, cannot be procured on board, feveral things may be ufed to ftop the ravages of fcorbutic ulcers, as claret or red port, vinegar, the vegetable aftringents alone, or combined with red wine, fruit of different forts, as oranges, tamarinds, &c. the recent root of the *caffavi*, the excellent effects of which I have already remarked; and I have no doubt that apples, plumbs, &c. as well as turnips, carrots, potatoes*, &c. grated into a pulp, and applied as a poultice to fores yielding a putrid difcharge, would have an effect fimilar to that of the frefh vegetable acid.

In

* A feaman who had ferved on board His Majefty's fhip Rainbow informed me, that he had been cured of the fcurvy in a high degree, on board and at fea, by eating a few raw potatoes

In June, 1783, I was ordered by Commiſſioner Laforey at Antigua to take charge of thirty-two invalids (all of whom had ulcers of long ſtanding, ſome in a ſloughy condition, and others with caries of the tibia) embarked on board a tranſport bound for England. I had, by repreſentation, obtained an order to have wine in the room of ſpirits, and I took care to be well ſupplied with ſugar, rice, ſago, limes, oranges, preſerved tamarinds, &c. When the ſtock of limes was expended, I was obliged to have recourſe to oranges cut in ſlices, and applied as a dreſſing; and though they became ſaturated with the putrid diſcharge much ſooner than the limes, yet I found them anſwer much better than any other thing within my reach. As our paſſage, however, was rather long, the oranges were alſo expended; I had then recourſe to a mixture of the ſyrup and pulp of tamarinds in French wine, and found it to anpotatoes every day — a practice which, he ſaid, had been recommended to the ſurgeon of the ſhip by an old American pilot. I have ſince inquired into the truth of this ſtory, and, from what I can learn, believe it to be a fact *.

* The good effects of raw potatoes, in the cure of the ſcurvy at ſea, have not eſcaped the attention of Dr. Blane in the work mentioned in the preceding note. — Editor.

ſwer very well in foul, ſloughy ſores as a detergent and antiſeptic. It may well be ſuppoſed that I could not gain much ground in the cure of theſe old ulcers while at ſea; indeed I was well ſatisfied with landing them all at Portſmouth in a better condition than I had received them.

The encomiums I have beſtowed on the external effects of lime juice, may, to many perſons, appear extravagant; but I hope they will not condemn me before they have made trial of the practice I recommend. In the mean time, I hope the following quotation of that marine Hippocrates, Dr. Lind, from Bontius, (the juſtneſs of which I have had many opportunities of obſerving) will plead in my behalf: —

"The moſt knowing practitioners in India "place greater confidence in lemons againſt "the malignant diſeaſes, peſtilential fevers, "&c. of the country, than in coſtly bezoar "or theriac. For my own part," ſays he, "I "affirm, that in my practice there I found "more benefit from them than from any one "ſingle remedy."

*Lothbury*,

October 5, 1785.

